# Research progress in the effects of terahertz waves on biomacromolecules

**DOI:** 10.1186/s40779-021-00321-8

**Published:** 2021-04-25

**Authors:** Liu Sun, Li Zhao, Rui-Yun Peng

**Affiliations:** Beijing Institute of Radiation Medicine, Haidian District, 27 Taiping Road, Beijing, 100850 China

**Keywords:** Terahertz waves, Biomacromolecules, Effect, Review

## Abstract

With the rapid development of terahertz technologies, basic research and applications of terahertz waves in biomedicine have attracted increasing attention. The rotation and vibrational energy levels of biomacromolecules fall in the energy range of terahertz waves; thus, terahertz waves might interact with biomacromolecules. Therefore, terahertz waves have been widely applied to explore features of the terahertz spectrum of biomacromolecules. However, the effects of terahertz waves on biomacromolecules are largely unexplored. Although some progress has been reported, there are still numerous technical barriers to clarifying the relation between terahertz waves and biomacromolecules and to realizing the accurate regulation of biological macromolecules by terahertz waves. Therefore, further investigations should be conducted in the future. In this paper, we reviewed terahertz waves and their biomedical research advantages, applications of terahertz waves on biomacromolecules and the effects of terahertz waves on biomacromolecules. These findings will provide novel ideas and methods for the research and application of terahertz waves in the biomedical field.

## Background

In past decades, terahertz technology has been widely used in various fields, such as aerospace, communications, security, and biomedicine. Importantly, the rotational and vibrational energy levels of biomacromolecules overlap with those of terahertz waves, indicating that terahertz waves might directly affect biomacromolecules. Therefore, it is valuable to clarify their relationship, which might result in a major breakthrough in biomedicine. Recently, numerous studies have focused on the interactions between terahertz waves and biomacromolecules. On the one hand, several groups have tried to explore the spectroscopic and structural properties of biomacromolecules under the frequency of terahertz waves. On the other hand, researchers have focused on the effects of terahertz waves on the expression and activity of biomacromolecules. In this paper, we reviewed progress in terahertz technology and the applications and the effects of terahertz waves on biomacromolecules.

## Terahertz waves in biomedical research

**Fig. 1:**
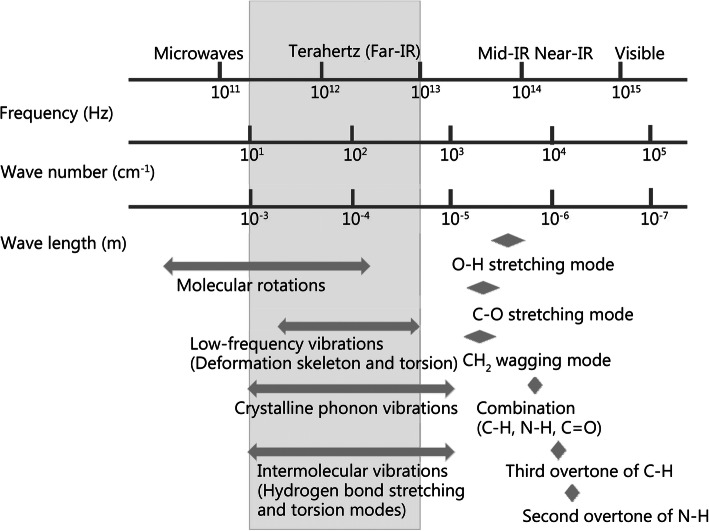
Terahertz band in the electromagnetic spectrum and corresponding molecular transitions. Infrared bands include far-infrared, Mid-infrared and near-infrared. Mid-infrared spectroscopy measures the vibration patterns of bending and stretching motions (such as C-O, C=O). Some molecular rotations, skeleton vibrations of macromolecules, weak intermolecular interaction and crystalline phonon vibrations *etc* correspond to terahertz bands. IR. Infrared

Terahertz waves refer to electromagnetic waves with frequencies ranging from 0.1 THz to 10 THz, with wavelengths from 30 μm to 3 mm, and vibration cycles from 0.1 ps to 10 ps [1]. Therefore, terahertz waves belong to the far-infrared spectrum in the field of radiophysics and are also defined as submillimeters lying between millimeter waves and infrared light in the field of optics, as shown in Fig. 1 [2].

Terahertz waves have been widely used in various fields, such as aerospace, communications, and medical applications, due to their unique physical properties. In the biomedical field, terahertz waves show potential in the following applications, as described below. 1) Fingerprint spectrality. The wavelength of terahertz waves is shorter than that of microwaves, which could produce a higher spatial resolution. The energy levels of vibrational, rotational, and weak intermolecular forces of organic macromolecules, such as hydrogen bonds and van der Waals forces, are mostly within the terahertz spectrum. These unique spectral properties can be used to resolve biomolecules and conformational characteristics [3]. A unique terahertz spectral fingerprint might be defined for each biomacromolecule [4]. Therefore, terahertz waves have the potential to be developed as a much more effective tool for analyzing the composition, structure and function of biomacromolecules. 2) Water absorption. The terahertz wave is a powerful biomedical testing tool to analyze the hydration state of biomolecules and to perform high-contrast imaging of biological tissues due to its strong water absorption capacity [5]. 3) Transience. Typical picosecond optical pulse terahertz waves can be used to analyze the transient absorption spectra of semiconductors and liquids due to their lower background noise [6]. Moreover, the terahertz wave and vibration of the protein structural domain fall on the same time scale, so they can reflect the transient characteristics of macromolecules [7]. 4) Coherence. Coherent measurement of terahertz pulses can directly measure the amplitude and phase and extract the refractive index and absorption parameters to improve the accuracy and reliability [8]. 5) Perspective. Terahertz waves can be applied for perspective imaging of opaque objects, attributed to their strong penetration of both dielectric materials and nonpolar liquids. 6) Broadband. The picosecond optical pulse of a terahertz wave results in a wide electromagnetic frequency band ranging from GHz to tens of THz. Therefore, it can be used to analyze the absorption spectrum of complex biological samples, as well as radio communication [6]. 7) Directionality. The wavelength of terahertz waves is longer than that of optical and near infrared spectroscopy, which reduces scattering during biological sample analysis [4].

## Applications of terahertz wave on biomacromolecules

The energies of the rotational and vibrational spectra of proteins, DNA, and RNA are (5.0–25.0) meV, and the rotational energy is (25.0–1000.0) meV, which is mostly in the energy level of the terahertz spectrum (1.2–83.0) meV. Researchers have applied terahertz spectroscopy to analyze the structural characteristics of biological macromolecules. Interpretation of the space conformation of macromolecules by terahertz spectroscopy will be beneficial for clarifying the molecular mechanisms.

### Detection of proteins by terahertz waves

Proteins, the most abundant biomacromolecules, are direct executors of life activities. The structural basis of the protein is a series of noncovalent bonds, such as hydrophobic bonds, hydrogen bonds and electrostatic forces, which are sensitive to terahertz waves. Amino acids, the basic units of proteins, also have resonance absorption peaks in the terahertz regime [9]. Moreover, the proteins in the biological tissues could form water shell structures with water by hydrogen bonds [10], which further enhances the absorption of the terahertz wave.

The terahertz wave spectrum has been used to analyze the conformational transformation of proteins and obtain abundant structural information. 1) Molecular vibrational model. The functions and activities of proteins are largely dependent on specific structures and space conformations. When a protein is excited by terahertz waves, its vibrational properties are altered, resulting in corresponding changes in the absorption spectrum [5]. Therefore, the dynamic vibrational mode of the protein could be monitored by using the terahertz spectrum detection method [5, 11]. 2) Resonance absorption peaks. Amino acids always have a universal structural formula, R-Cα(H)(NH_3_^+^)-COO^−^, in which R represents a variable side chain. Variable side chains play important roles in maintaining the dielectric and electronic properties of proteins [12]. The interaction between terahertz radiation and amino acid solution is mainly through molecular rotational and vibrational modes. The weak intermolecular forces, skeletal deformation vibrations, vibration–rotation transition dipoles, and low-frequency vibration absorption of the crystal lattice responded in different positions and intensities of the terahertz wave [13]. For example, different amino acids have different peak positions in the terahertz spectra [14–20].

The theoretical model suggested that terahertz wave radiation did not cause rupture or reconstruction of chemical covalent bonds. However, it could excite the rotational energy level of proteins to alter the spatial conformation and might influence the interactions between proteins.

### Detection of nucleic acids by terahertz waves

Nucleic acids are macromolecules that store the genetic information of life [21]. The energy levels of vibrational and rotational excitation, which are associated with the structure of nucleic acids, were within the terahertz spectrum [5]. Therefore, terahertz waves are potential and sensitive tools to identify and analyze physical and chemical processes during the synthesis and catabolism of nucleic acids. The terahertz absorption spectra of the five main bases are different from each other, and the same absorption peak represents a shared base. The nucleoside absorption peak mainly originates from hydrogen bonds, which link pentose and pentose, pentose and base, base and base [21]. Nucleic acids can be divided into two groups: DNA, which is mainly located in the nucleus, and RNA, which is always distributed in the cytoplasm.

DNA, the carrier, transmitter and the material basis of genetic information, determines the genotype of individuals. Terahertz waves are sensitive to the alteration of DNA structures mainly through a collective vibrational mode, which is determined by molecular configuration and spatial conformation. The difference in the dielectric properties of the four bases was attributed to the intermolecular movement of hydrogen bonds, and bending of one hydrogen bond would affect the torsion of another hydrogen bond [22].

The dipole-dipole interactions between C and G and between A and T are the physicochemical basis for DNA replication. The dipole moments of A and T and C and G are equal, and the directions are opposite. Therefore, C must be paired with G, and A must be paired with T when DNA replication and repair occur. All genetic information is stored in DNA by the order of paired bases. Analog computation of the DNA replication state suggested that the spectrum of the replicating DNA molecules was located in the terahertz regime [23].

Terahertz spectroscopy could distinguish the absorption of different DNA molecules. Therefore, non-labelled detection could be used to detect DNA molecules by acquiring the absorptive and refractive indices [4]. The composition and topology of DNA molecules are very sensitive to the movement of hydrogen bonds, which strongly depended on the double-stranded base pair. The vibrational mode of the hydrogen bond in the -NH_2_ of the complementary DNA contributed to the responses of DNA to terahertz waves. Studies on the terahertz transmission spectrum and resonance frequency of DNA molecules showed that a greater difference in DNA sequences resulted in a lower terahertz transmittance and a lower resonance frequency [5]. Studies also showed that the refractive index of hybridized DNA was higher than that of denatured DNA, suggesting the potential application of terahertz technology in analyzing the binding state of oligomeric or polymeric nuclear acids [24]. Recently, it has been reported that DNA methylation can be detected by terahertz waves with a spectral frequency of 1.67 THz, which was in good agreement with the gold standard enzyme-linked immunosorbent assay [25, 26].

RNA plays a decisive role in the translation and expression of genetic information, which are crucial in biological evolution. However, to our knowledge, there are still no publications on the application of terahertz spectroscopy for RNA detection.

### Detection of terahertz wave on saccharides

Polysaccharides, carbohydrates with complex molecular structures, are formed by condensation and dehydration of many monosaccharide molecules. Polysaccharides are important components of organisms and can provide energy and carbon sources to support survival. Saccharides showed distinct terahertz spectrum characteristics. Saccharides contain a large number of hydrogen atoms and oxygen atoms, and numerous hydrogen bonds can be formed by themselves or with other biomolecules. It has been demonstrated that saccharides show a distinct terahertz absorption spectrum due to their structural characteristics. The terahertz absorption spectrum of saccharides was directly related to the vibration of intermolecular hydrogen bonds [5]. Ma et al. [27] detected the absorption spectra of D-(−)-ribose, D-glucose, α-lactose monohydrate and β-lactose. The absorption of different sugars in the measured band was significantly different. Chen et al. [28] used terahertz time-domain spectroscopy to study the terahertz absorption spectra of D-glucose and lactose monohydrate, two typical monosaccharides and disaccharides with similar structures. These researchers found that lactose monohydrate had three absorption peaks at 0.53, 1.19 and 1.38 THz, while D-glucose only had one absorption peak at 1.44 THz, suggesting that lactose monohydrate and D-glucose presented different terahertz fingerprint absorption characteristics. Therefore, the THz time-domain spectroscopy technique is a sensitive tool to analyze the structural alteration of carbohydrate molecules.

### Detection of terahertz wave on lipids

Lipids can be classified into three groups: fats, lipids (phospholipids) and sterols (cholesterol, sex hormones and vitamin D). Lipids regulate various life processes, such as energy conversion, material transport, information recognition and transmission, and cell development and differentiation [29].

Research on the terahertz spectrum of lipids is deficient. Zhang et al. [30] reported that terahertz time-domain spectroscopy technology would be beneficial for the early diagnosis of cardiovascular and cerebrovascular diseases. It has been widely demonstrated that the rupture of unstable atherosclerotic plaques with lipid cores is the main cause of acute cardiovascular and cerebrovascular diseases. Cholesterol crystals, the main component of the lipid core, showed a unique terahertz spectrum and could be distinguished from normal cell structures.

## Effects of terahertz wave on biomacromolecules

Generally, it is believed that THz waves can strongly affect organisms due to their biophysical properties and unique responses in organisms [31, 32]. Therefore, the effects of terahertz waves on organisms might be mediated by biological macromolecules. Briefly, terahertz waves altered the space conformation of biomacromolecules by exciting rotational energy levels, which in turn influenced the effects on biomacromolecules and finally resulted in structural and functional changes in organisms. Therefore, terahertz waves are a potential tool to analyze, identify, precisely manipulate and regulate the structures and properties of biological macromolecules [31–34].

Until now, research on the effects of terahertz waves on biomacromolecules has faced major technical challenges. Most of the studies have focused on biomacromolecules, such as proteins, nucleic acids and polysaccharides. Research has mainly focused on the molecular mechanisms underlying the effects of terahertz waves on organisms [35–37]. Mechanistic studies provide strong support for exploring the roles of biomacromolecules in organisms.

### Effect of terahertz waves on proteins

The effects of terahertz waves on biomacromolecules depend on various factors, such as the radiation source, radiation intensity and frequency, radiation duration, and structural and functional differences of biomacromolecules. Most of the studies suggest that terahertz radiation can only affect biological macromolecules under high power, long-term exposure and at a specific frequency.

Studies have reported that terahertz wave radiation alters the activity and expression of proteins, which is closely associated with radiation intensity [38]. Titova et al. [39] found that the levels of tumor suppressor proteins, cell cycle regulatory proteins, and damage repair proteins were upregulated in artificial human skin tissues after exposure to terahertz waves with a strong pulse of (0.1–1.0) μJ. Ten minutes after exposure, the phosphorylation activity of histone H2AX was increased with the upregulated expression of the apoptosis-related proteins p53 and KU70 and the cell cycle regulatory proteins p21, p16, p15 and p27. These data indicated that DNA damage was induced after exposure and that the repair mechanisms were rapidly activated. Homenko et al. [40] reported that the activity of alkaline phosphatase was significantly reduced in endothelial cells after exposure to 0.10 THz for 2 h. Borovkova et al. [41] showed that after continuous terahertz radiation at (0.12–0.18) THz with an average power density of 3.2 mW/cm^2^ for 5 min, the space conformation of membrane proteins was changed, several apoptotic proteins were released from mitochondria to cytoplasm, and proteolytic enzymes were also released from lysosomes in the glial cell line C6. Moreover, there was a large number of aquaporins in glial cells, and terahertz radiation could induce conformational alteration of aquaporins, which resulted in enhanced water inflow and membrane deformation. Demidova et al. [42] discovered that green fluorescent protein expression was induced in *Escherichia coli* after exposure for 15 min under constant terahertz radiation at 1.4 W/cm^2^. Chen et al. [43] revealed that C57BL/6 J mouse skin tissue interleukin-1beta (IL-1β), interleukin-6 (IL-6) and tumor necrosis factor-α (TNF-α) was significantly increased after exposure to 0.22 THz with an average power density of 50 mW/cm^2^ for 5 min. This suggested terahertz waves could trigger an inflammatory response. Tan et al. [44] found terahertz waves downregulated synuclein (SYN) expression in primary hippocampal neurons and downregulated postsynaptic density protein 95 (PSD-95) expression in primary cortical neurons, indicating different cells expressed different proteins by terahertz waves.

Other groups reported that terahertz waves had no obvious effects on the expression and activity of proteins. De Amicis et al. [45] found that low-frequency terahertz waves from a free electron laser (FEL) did not alter the expression of histone H2AX and pivotal proteins in apoptosis-inducing and survival-promoting signaling pathways in fetal fibroblasts, which indicated that no significant DNA damage was induced. It was revealed that broadband coherent terahertz waves at 0.5 THz with an average power of 1.2 mW produced no obvious influence on cell attachment, morphology, proliferation or differentiation in either human epithelial cells or embryonic stem cells. Moreover, terahertz could not alter the conformation of adsorbed proteins, which might be attributed to the adaptive capacity to terahertz wave exposure [46]. Homenko et al. [40] found that the activity of immobilized alkaline phosphatase was not changed after exposure to 0.10 THz terahertz radiation for 2 h. Koyama et al. [47] also reported that a 5 mW/cm^2^ terahertz wave at 0.12 THz did not influence heat shock protein expression in human corneal epithelial cells (HCE-T).

### Effects of terahertz waves on nucleic acids

Consistent with that on proteins, the effects of terahertz waves on DNA and RNA were determined by radiation sources, radiation frequency, intensity and duration.

At the DNA level, it has been reported that specific terahertz waves interfere with DNA replication and gene expression [3]. Under certain conditions, linear and nonlinear interactions between the terahertz electromagnetic field and DNA resonance might significantly alter DNA replication and synthesis and even induce local bubbles in the DNA strands [48]. Hintzsche et al. [49] found that 0.106 THz terahertz waves prevented the separation of chromatids during anaphase and telophase of mitosis in hamster hybrid cells. Berns et al. [50] showed that exposure to 1.5 THz FEL at 100 pulses directly reduced absorption in DNA molecules and inhibited DNA synthesis in mammalian cells. Studies from Cheon et al. [51] suggested that 1.7 THz high-power terahertz exposure resulted in DNA demethylation in hematological tumor cells. Titova et al. [39] revealed that terahertz radiation caused DNA damage in human skin tissue at a strong terahertz pulse of 1.0 μJ and a repetition rate of 1 kHz. The tubulin beta 3 (Tubb3) and synaptophysin (SYP) genes were downregulated by 0.22 THz and 50 mW /cm^2^ irradiation of Neuro-2a cells, suggesting that terahertz waves could inhibit synaptic growth [52]. Lu et al. [53] radiated C57BL/6 J mouse retinal tissue at the average power density of 80 mW/cm^2^. They observed that terahertz waves could lead to abnormal retinal gene expression and tissue damage after 2 min of radiation.

Studies from other groups indicated that terahertz waves have no obvious effect on the structures and functions of DNA. Wei et al. [54] discovered that exposure to (0.1–3.0) THz terahertz wave for 60 min, with an average intensity of 60 W/cm^2^, could not damage the integrity of DNA in spermatozoa. Similar results were reported by Wilmink et al. [55], who used a gas-phase terahertz laser to radiate human skin fibroblasts for 80 min at 2.52 THz in a temperature-controlled chamber. Zeni et al. [56] exposed human peripheral blood leukocytes to 0.12 THz and 0.13 THz terahertz waves for 20 min, and no detectable DNA damage was observed. Hintzsche et al. [49] also revealed that human skin fibroblasts and human hamster hybrid cell lines were not sensitive to 0.106 THz terahertz waves, and no DNA strand break was detected under power densities between 0 mW/cm^2^ and 2 mW/cm^2^ for 2, 8 and 24 h.

At the RNA level, a specific terahertz wave could affect gene expression. Alexandrov et al. [57] radiated mouse pluripotent stem cells with broadband terahertz waves at 10 THz. These researchers found that terahertz waves promoted the differentiation of mouse pluripotent stem cells after 12 h of radiation by upregulating the expression of peroxisome proliferator-activated receptor gamma, adiponectin, glucose transporter 4 and human adipocyte fatty acids. Zhao et al. [58] used a wide-band terahertz source to radiate three different types of cells, epithelial cells, human corneal epithelial cells and human M lymphocytes, under an 800 nm pulse with a repetition rate of 1 kHz, an average power of 1 mW and a peak power of up to 2 mW. Six hours after radiation, the expression of numerous genes, such as proto-oncogenes and anaplastic lymphoma kinase (ALK), was upregulated. Interestingly, only human centromere protein E expression was downregulated in human corneal epithelial cells. Titova et al. [59] exposed artificial skin tissue to (0.20–0.25) THz, and energy density per pulse was 6 μJ/cm^2^ for 0.1 μJ THz pulses and 60 μJ/cm^2^ for 1 μJ THz pulses. Tissue-specific alterations in gene expression were observed. For example, squamous cell carcinoma-associated genes, such as calcitonin, recombinant human small proline-rich protein-1B, and recombinant human involucrin protein, decreased obviously after radiation. Moreover, Bock et al. [60] used broadband terahertz waves at 10 THz in the center to radiate mouse mesenchymal stem cells (MSCs). Generally, no significant differential expression could be detected in 89% of the genes, while the expression of the other 11% of genes was activated or inhibited. The effects of terahertz waves on gene expression were closely related to radiation conditions. For example, exposure to terahertz waves for 9 h could significantly alter gene expression in MSCs, including increased expression of the transcription factors peroxisome proliferator-activated receptor γ, adiponectin, glucose transporter 4 and human adipocyte-type fatty acid binding protein 4 and decreased expression of the nuclear receptor transcription factor liver-X-receptor (LXR). Terahertz radiation accelerated chondrocyte differentiation of MSCs. In addition, several studies showed that THz waves did not produce obvious effects on RNA. Bogomazova et al. [61] exposed human embryonic stem cells to 2.3 THz narrowband terahertz waves at an average power density of 1.4 W/cm^2^ and controlled temperature strictly. They explored whether the expression of phosphorylated histone H2AX (γH2AX) was altered after radiation for 2 h.

### Effects of terahertz wave on lipids

In addition to proteins and nucleic acids, other biomacromolecules, such as lipids, are affected by terahertz waves. Ramundo et al. [62] found that the permeability of liposomes that contain a carbonic anhydride cation increased after radiation with 0.13 THz pulsed terahertz waves. However, related studies are lacking.

## Prospects and challenges

Terahertz technology is an important interdisciplinary frontier field that provides unprecedented opportunities to promote technological innovation, economic development and national security. Terahertz waves strongly interact with biomacromolecules, and the terahertz spectrum of biomacromolecules contains numerous physical and chemical information. Therefore, the development of terahertz technologies has attracted increasing attention in biomedical fields. However, basic research and the application of terahertz technologies in the biomedicine field are in the initial phase. A number of scientific problems and technological barriers need to be addressed, such as the distinct sensitivity of different biomacromolecules to terahertz waves and the roles of terahertz parameters on the effects of biomacromolecules. Moreover, the mechanisms of interactions between terahertz waves and biomolecules are still unknown. The primary object for applying terahertz technology in the biomedical field is to illuminate biological phenomena by revealing the basic rules of interactions between macromolecules and macromolecules, as well as cells and cells.

However, there are still several problems and challenges that need to be solved, some of which are presented as follows. 1) Temperature. During the detection of biological samples, terahertz waves cause biological responses that are sensitive to temperature, and the temperature difference should not exceed 0.1 °C. 2) Water. Terahertz waves could be strongly absorbed by water. It has been demonstrated that water vapor in the air and the solution of biological macromolecules always cause loud noises, resulting in poor sensitivity and weak terahertz spectrum signals. Therefore, it is necessary to reduce water vapor in the air before radiation, such as by injecting N_2_ gas. Moreover, the amount of culture fluid should be reduced to the minimum volume. 3) Samples. Genetic materials, such as DNA and RNA, are isolated from organisms, and the amount is always extremely limited. Biological samples are also easy to denature. 4) Resolution. Compared to X-rays, γ-rays and visible light, terahertz waves have longer wavelengths. The resolution of the detection system is inversely related to the wavelength of the emitted wave. Therefore, the wavelength of terahertz waves limits the spatial resolution in imaging systems. Several strategies have been tested to improve the spatial resolution, such as insertion of small holes.

## Conclusion

The interaction between terahertz and biomacromolecules is very important. To date, we have found that different biomacromolecules have different absorption peaks. However, no complete spectral analysis of biomacromolecules has been established. In addition, terahertz waves can cause structural changes in some biological macromolecules, but the frequency range has not been clarified, and the ultimate function of organisms has not been explained. Although research on the relationship between terahertz waves and biological macromolecules is still in its infancy, we believe that major breakthroughs will be realized in the near future with the efforts of professional scholars and the application of advanced terahertz instruments.

## Data Availability

All data generated or analyzed during this study are included in this published review.

## Abbreviations

ALK: Anaplastic lymphoma kinase
FEL: Free electron laser
H2AX: Histone H2AX
HCE-T: Human corneal epithelial cells
IL-1β: Interleukin-1beta
IL-6: Interleukin-6
LXR: Liver-X-receptor
MSCs: Mesenchymal stem cells
PSD-95: Postsynaptic density protein 95
SYN: Synuclein
TNF-α: Tumor necrosis factor
γH2AX: Phosphorylated histone H2AX
